# Neonatal healthcare professionals’ experiences of intact cord resuscitation in the mother´s bed- an interview study

**DOI:** 10.1186/s12884-024-06558-0

**Published:** 2024-05-15

**Authors:** Katarina Patriksson, Ola Andersson, Li Thies-Lagergren, Maria Rönnerhag

**Affiliations:** 1 Department of Health Sciences, University West, Trollhättan, SE-46186 Sweden; 2 Department of Paediatrics/Neonatology, NU-Hospital Group, Trollhättan, SE-46185 Sweden; 3 Department of Clinical Sciences Lund, Paediatrics/Neonatology, Lund University, Lund, SE-221 84 Sweden; 4 Department of Neonatology, Skåne University Hospital, Malmö, Lund, SE-222 42 Sweden; 5 Department of Health Science, Midwifery Research - Reproductive, Perinatal and Sexual Health, Lund University, Lund, SE-22100 Sweden

**Keywords:** Childbirth, Intact cord, Neonatal care, Resuscitation, Qualitative interview

## Abstract

**Background:**

Intact cord resuscitation in the first three minutes of life improves oxygenation and Apgar scores. The practise of intact cord resuscitation implies the umbilical cord still being connected to the placenta for at least one minute while providing temperature control and equipment for resuscitation. Healthcare professionals described practical challenges in providing intact cord resuscitation. This study aimed to explore neonatal healthcare professionals’ experiences of providing intact cord resuscitation in the mother’s bed.

**Method:**

An interview study with an inductive, interpretative approach was chosen and analysed according to reflexive thematic analysis by Braun & Clarke. An open interview guide was used and 20 individual interviews with neonatal healthcare professionals were performed*.* The study was conducted at five level I-III neonatal care units. In Sweden, resuscitation is performed either in or outside the labour room.

**Results:**

The results contributed insight into the participants’ experiences of prerequisites for providing neonatal care in intact cord resuscitation. The sense of the mother’s vulnerability was noticeable, as the participants reported reducing the risk of exposure to protect and preserve the mother’s integrity. The practical challenges in the environment involved working in a limited space. The desire for multi-professional team training comprised education and training as well as debriefing to manage intact cord resuscitation.

**Conclusion:**

The result of the present study highlights the fact that neonatal healthcare professionals’ experiences of providing ICR in the mother’s bed were positive and had significant benefits for the neonate, namely zero separation between the neonate and parents and better physical recovery for the neonate. However, the fact that ICR in the mother’s bed can be challenging in several ways, such as emotionally, managing environmental circumstances and ensuring effective team collaboration. Therefore, it is of the utmost importance that healthcare professionals are given the opportunity to reflect and train together as a team. Future recommendations are to summarize evidence-based knowledge to design guidelines for ICR situation.

## Background

Approximately 5–10% of neonates worldwide need assistance with breathing after childbirth [1]. Although adverse perinatal outcomes are uncommon, approximately every 10th neonate in Sweden requires assistance to establish independent breathing after childbirth and 1% need extensive medical interventions (The Swedish National Board of Health and Welfare [NBHW], [2] defined as neonatal cardiopulmonary resuscitation [3]. Childbirth can become life-threatening when it involves risks and complications for the neonate, such as birth asphyxia, which is caused by impaired gas exchange and can occur before, during, or after birth [4]. Complications leading to birth asphyxia include placenta abruption, uterine rupture, tight nuchal cord, cord prolapse, or infection [5]. Other factors that can cause suspected birth asphyxia are incautious use of synthetic oxytocin, shoulder dystocia, or a difficult breech birth, which commonly leads to immediate cutting of the umbilical cord and starting resuscitation [6].

Until 2008, the routine for non-vigorous neonates in most Swedish labour wards was to clamp the umbilical cord within 30 s (early cord clamping) after childbirth [7]. Evidence demonstrates that early cord clamping is associated with lower Apgar scores and increased risk of intraventricular haemorrhage after preterm birth, leading to a higher risk of prolonged asphyxia and permanent impairment for the neonate [8]. Andersson et al. [9] reported that intact cord resuscitation (ICR) in the first three minutes of life improved oxygenation and Apgar scores during the first ten minutes of life in late preterm and term neonates. Regardless of birth mode, it is feasible to perform resuscitation on neonates during sustained cord circulation, clamping the umbilical cord only when the neonate is physiologically ready [10]. The practise of ICR implies the umbilical cord still being connected to the placenta for at least one minute while providing temperature control and equipment for resuscitation [11]. More advanced resuscitation including early and continuous positive pressure ventilation and/or intubation, minimizing hypoxia followed by acidosis and maintaining normal temperature are strategies to potentially reduce neonate mortality [4].

It has been described that changing practises can face obstacles within healthcare [12]. International studies have described that healthcare professionals described practical challenges in providing ICR, as well as concerns about having the resuscitation equipment available in a timely manner. Other challenges mentioned were the integration of ICR into a clinical routine and team training, as well as providing ICR beside the mother with a non-vigorous neonate while witnessed by parents [13]. Thus, neonatal interventions can be challenging in various ways. To the best of our knowledge, there are few previous studies on neonatal healthcare professionals’ experiences of providing ICR in the mother’s bed. To narrow this knowledge gap and obtain a more comprehensive view of this issue, it is essential to illuminate neonatal healthcare professionals’ experiences of practicing ICR.

This is important as neonatal healthcare professionals caring for a non-vigorous neonate are obliged to work according to best evidence. This interview study aims to explore neonatal healthcare professionals’ experiences of providing intact cord resuscitation in the mother’s bed.

## Method

### Design

This qualitative study is part of a randomized control multi-centre trial entitled the Sustained cord circulation And Ventilation (SAVE) study aimed at implementing and evaluating ICR for all neonates born vaginally in Sweden [14]. A qualitative study design was chosen to explore, analyse and identify patterns [15]. The study design was based on an inductive, interpretative approach and analysed according to reflexive thematic analysis by Braun & Clark [16] and was deemed appropriate to address the study aim. Braun & Clarke [16] emphasize the importance of exploring participants’ individual interpretations of their reality to understand the rationale behind behaviours in professional practice. In the present study, the intention was to interpret and understand aspects of the participants’ experiences of ICR in the mother’s bed in their professional practice.

### Participants and setting

A purposive sampling was used to carefully collect relevant data [16]. The participants consisted of 20 healthcare professionals working in neonatal care including paediatric nurses, regional nurses, midwives, and physicians (Table 1). Although the participants included both women and men, the majority were women. All participants were Swedish-speaking and aged between 27 and 54 years (mean 42). Their overall work experience ranged between one year and 30 years, while their neonatal care experience ranged between one year and 28 years. One of the participants only served on the neonatal care unit when on-call. The inclusion criterion was that the participants had practised ICR in the mother’s bed.

**Table 1:** Overview of the participants

Nr	Profession	Number of years in profession
1	Physician	22
2	Paediatric nurse	18
3	Physician	10
4	Midwife	17
5	Nurse	10
6	Intensive care nurse	10
7	Paediatric Nurse	28
8	Paediatric Nurse	28
9	Paediatric Nurse	14
10	Physician	1
11	Physician	28
12	Physician	9
13	Paediatric Nurse	28
14	Paediatric Nurse	21
15	Paediatric Nurse	22
16	Paediatric Nurse	30
17	Intensive care Nurse	29
18	Nurse	3
19	Paediatric Nurse	7
20	Physician	3

The study was conducted at five level I-III neonatal care units, including a university hospital with a level III Neonatal Intensive Care Unit (NICU). The number of neonatal healthcare professionals employed at the five units ranged from 45–170. In some hospitals in Sweden, neonates are resuscitated in the labour room, while in others the neonate is taken to another room. When the neonate is taken outside the labour room, the other parent usually accompanies the neonate. In ICR in the mother’s bed, neonatal healthcare professionals work together with obstetric healthcare professionals in the labour room.

### Procedures

Data were collected between October 2021 and April 2022. An open interview guide was used, and 20 individual interviews were performed with focus on the aim of the present study. The majority, namely 17 interviews, were performed using the Zoom media tool, which enables the informant and interviewer to see each other during the interview by means of a camera. The remaining three interviews were conducted in person. The study group, including 20 participants, was considered homogenous due to all performing ICR and the interviews resulted in a rich material [17]. All interviews were collected by the first author K.P. of present study. The opening question was: *Can you please share your experiences of neonatal cardiopulmonary resuscitation of a neonate including ICR in the mother’s bed.* An example of a probing follow-up question was: *Can you please tell me more about that?* Each interview lasted between 20 and 40 min (mean 31). The audio recordings were transcribed verbatim.

## Analysis

A qualitative reflexive thematic analysis by Braun & Clarke [16] was chosen as it provides a meaningful amount of data through a systematic repetitive process including a back-and-forth movement throughout the six phases [16]. The first phase involved verbatim transcription of the interviews by K.P. The interview text was then analysed by K.P. and M.R and further validated by LTL. The text was read in its entirety to gain familiarity with the content and initial reflections were noted. In the second phase, statements relevant to the aim were coded. The third phase consisted of searching for sub-themes. All data relevant to the aim including similarities and differences were grouped into sub-themes based on the code’s consensus. The fourth phase involved reviewing the sub-themes through further analysis to verify how the data related to each other. The characteristics of each sub-theme were defined and named. The sixth and final phase concerned describing and labelling the overarching theme. A thematic structure of sub-themes and themes emerged through the analysis process, which constitutes an interpretation of the participants’ experiences of ICR in the mother’s bed.

## Ethical considerations

Ethical approval and permission to conduct the study were obtained from the Swedish Ethical Review Authority (Dnr 2021–03688). The study complied with internationally accepted ethical principles for medical research involving human subjects, designed to protect individuals and ensure respect for human dignity [18]. All participants gave their informed consent. According to the European General Data Protection Regulation (GDPR), the informed consent must be documented, specify a clear purpose, be voluntary, time-limited, easy to understand and possible to withdraw without any explanation [19].

## Results

The results highlighted both positive and negative experiences of providing ICR among healthcare professionals within neonatal care. It emerged that the healthcare professionals experienced psychological och practical challenges. With a total of three themes and eight sub-themes emerged from the qualitative data analysis, as presented in Table 2. Each sub-theme is strengthened by quotations from the participants.

**Table 2:** Overview of overarching theme with themes and sub**-**themes

Overarching theme	The prerequisites for providing neonatal care during intact cord resuscitation		
Themes	The sense of the mother's vulnerability	The practical challenges in the environment	The desire for multi-professional team training
Sub**-**themes	Handling emotions in an unfamiliar situation Preventing exposure and preserving dignity Promoting attachment between the mother and neonate	Managing intact cord resuscitation in a limited space Gaining access to the equipment	Sharing the same mindset Being vigilant about only adding team members when necessary Being prepared for intact umbilical cord resuscitation

## The prerequisites for providing neonatal care during intact cord resuscitation in the mother’s bed

### The sense of the mother's vulnerability

#### Handling emotions in an unfamiliar situation

This sub**-**theme describes participants’ emotions in an unfamiliar situation in terms of not being accustomed to ventilating the neonate when the mother was in a lithotomy position and the woman’s private body parts was exposed. The participants experienced concerns related to perform ICR in the mother’s bed, they were anxious about how they would react to being physically close to the mother. This due to body fluids such as blood and amniotic fluid, which some described as an unusual situation. They had to handle these feelings of concerns when performing ICR as they did not want to transfer them to the mother to avoid a negative childbirth experience in a vulnerable situation. One participant described the feeling of being liable in a new and unfamiliar situation due to the lithotomy positioning. This posed a risk of delayed initiation of ventilation of the neonate. Another experience reported by new employees in the neonatal field was that due to the implementation of the SAVE study, they could better focus on the neonate. Therefore, being close to the mother and work in the mother’s bed felt natural. Sometimes taking the neonate to a resuscitation table was easier as there were clean surfaces and items that were needed were within reach. However, at the same time, some participants reported that it was more natural to resuscitate the neonate in the mother’s bed as separation of the dyad was reduced. Finally, they expressed being on the right track. One participant stated that the parents had already given their informed consent to the SAVE study during maternal care and that the mother must cope with the situation regardless of the outcome. At the same time, participants admitted that before ICR it could be difficult to visualize the sequence of events for those involved. Participants expressed it as follows;

“Then you sometimes know that the mothers, yes if they poop during childbirth or so. I have never ended up in that situation. But I can imagine it and I am very convinced that I would never want to be a midwife. I'm not attracted to this gynaecological environment, but I do it anyway and I do not think I do a worse job” (N6)

“But it felt somehow nice anyway even if it was hard for us to stand there. Everyone was in the present but a little in their own way and had their own experience” (P10).

#### Preventing exposure and preserving dignity

This sub-theme highlights the fact that mothers were in vulnerable situations, especially when there was an increased number of interprofessional team members in the room. Therefore, it was of importance to preventing exposure and preserving the mother’s dignity. The participants expressed that the mother not always was aware of her vulnerability due to her feelings of happiness and the relief that the birth was over. In such a situation, the participants stated that midwives at the labour ward were often aware of the mother's vulnerability and put a towel over her lower abdomen to preventing exposure. On one occasion, the physician from the neonatal unit suggested that the midwife should put a towel over the mother. The physician was ventilating and could not put on a towel herself but expressed that she thought the mother was too exposed. The participants did not think of their own situation but expressed sympathy for the mother due to the large number of interprofessional team members in the labour room and the mother´s vulnerability caused by her exposure. This influenced on the mother's dignity from the perspectives of the participants. They stated that they would never want to be in the mother's exposed situation. They reflected that one day they might meet a mother who feels despaired and uncomfortable in this situation. The participants expressed;

“They put something over the mother's abdomen and then it felt more ok” (P5).

“Many were perhaps afraid of getting so close to the mother. How are we going to protect her because it will be quite intimate? In cases that have been reflected on, the midwife or assistant nurse put a towel over the mother´s abdomen” (P14).

#### Promoting attachment between the mother and neonate

This sub-theme describes the importance of not separating the neonate and the mother, but also the joy of being a part of this miracle when the parents receive a new family member. The participants wished that the mothers should have the opportunity to have the neonate skin-to-skin, which promotes attachment between the mother and neonate. Some mothers expressed to the participants that they appreciated the closeness to the neonate, yet they were not prepared to watch the ICR. Furthermore, they thought it was meaningful to hear the neonate, what was happening as well as the dialogue between the interprofessional team members. To promote attachment the participants stood and, for example, held a continuous positive airway pressure (CPAP) while the neonate lay skin-to-skin with the mother and the father stood next to them. The participants stated that when the father attended a resuscitation situation outside the delivery room, he appeared calmer than the mother, who was not present. The other parent standing at the mother's side was perceived as meaningful as the physician could explain to both parents at the same time what was happening and what was planned. One physician explained that it was a constant reminder to always be clear and tell the parents what was happening. When the neonate and parents were not separated, the participants felt satisfaction with their work. They observed that the father focused on the mother but could also see what was happening to their neonate. The participants expressed that the other parent became a visitor in the resuscitation room outside the labour room, but when both parents were still in the room during the ICR more involvement was seen. They also thought it was important that both parents were present as it enabled them to process what they had been through as a couple. One participant expressed that a mother in the labour room said that she drew the winning ticket, by which she meant that the neonate was not taken out of the room. A participant expressed;

“When we were done there, we placed the newborn on the mother’s chest and then I stood beside the bed to hold the CPAP and talk with the mother for a while and then we took the CPAP away. The only thing she talked about was that she was so lucky to draw the winning ticket” (N15).

“But one neonate who had a bit of a hard time in the beginning got CPAP and ended up on the mother’s chest, we looked again, and the neonate was still struggling. Then, we stood at the resuscitation table and gave CPAP to the neonate for an hour to try to avoid a separation” (P20).

### The practical challenges in the environment

#### Managing intact cord resuscitation in a limited space

This sub-theme described the limited space due to lithotomy positioning. One participant stated that after childbirth and before placental delivery there was sufficient space to reach the neonate and give ICR in the mother’s bed. This was possible when the mother was in a stable physical condition. Thus, the midwife could monitor the mother’s condition from a distance. While the participants needed to be in the labour room due to the neonate’s condition, the limited space sometimes made it difficult to see and use the necessary equipment. Standing at a resuscitation table bedside the mother was sometimes described as an advantage as it was easy to reach the neonate from several angles and the neonate could be kept warm. Participants felt safe in their role due to education or work experience and could adapt to different resuscitation situations. Participants expressed;

“There are many who want to be in the same place. The midwife or obstetrician want to be with the mother in the lithotomy position and that's exactly where I and the neonate are” (P1).

“Yes, but it was crowded or he was standing and you know the mother's legs, two physicians there and a CPAP and lots of people. So it was ohh and I felt that I did not have as much contact with the parents as I usually try to have” (P15).

#### Gaining access to the equipment

This sub-theme described the fact that it was often difficult to reach the neonate due to the placement of electrodes and pulse oximeter probes. The participants expressed that it was sometimes easier to gain access to the neonate by bending over the mother's legs. It was also easier to take care of the neonate when the delivery bed had the lower extra part. Participants expressed that it was sometimes difficult to put on the extra bed part under stressful conditions and it could be hard to find the equipment required in the resuscitation situation. They often had to make several attempts to reach the equipment, which was difficult as some of the interprofessional team members were standing in front of it. Participants expressed that the labour room was messy and noisy but there was always a will to help each other. If the neonate’s parameters could not be seen on the monitor some of the interprofessional team members in the room informed about them, which made all involved calmer. Participants expressed;

“Occasionally you feel that you require a couple of extra hands, but I had many extra hands that could help me if necessary” (P3).

“On one occasion it was difficult to access the monitor because there were people in the way. When I was going to take the stethoscope, it got stuck in the monitoring device and I could not access it. In the end, I gave up because by then the neonate was fine” (P12).

### The desire for interprofessional team training

#### Sharing the same mindset

This sub**-**theme concerns the interprofessional team members and their interpretation of the shared mindset of ICR. Some participants expressed that ICR in the mother’s bed has benefits for the neonate and they were positive about adapting to the neonatal intervention, while others were reluctant or hesitant. Hesitancy was related to uncertainty about whether ICR would be successful in terms of a healthier neonate and a positive outcome compared to the previous method when the umbilical cord was immediately cut and the neonate resuscitated outside the labour room. The participants experienced that they had different perspectives and views concerning how they interpreted the intention of ICR related to their various professions and specialties, as well as how clearly the purpose of ICR was communicated. There were contrasting views on how they valued different aspects of ICR. This was based on whether they took the perspective of the mother or the neonate and influenced interprofessional team collaboration. An experience that the participants agreed on was that debriefing before and after ICR was significant for a shared mindset. However, in some emergency situations it was a challenge to find the time for this before ICR. One participant expressed;

“(…) she said shouldn't we put the baby on the mother's chest? I was ventilating the child; therefore it was not very smart. I didn't feel like discussing it. Sometimes we don't really notice the same thing (…). I said we need a little more time. I said afterward that I promised to put the neonate on the mother’s chest when the neonate was physiologically ready. But she hadn't understood the situation like I had. She hadn't understood that I was ventilating the child” (P2).

#### Being vigilant about only adding team members when necessary

This sub**-**theme focuses on an adequate number of interprofessional team members in relation to the importance of vigilant team leadership. Participants experienced that the team consisted of too many members. However, adding members was a way to ensure that the team had the requisite skills and desired diversity, as well as an opportunity for new healthcare professionals to learn ICR in the mother’s bed. Nevertheless, an increase in a larger team had consequences such as poor communication, fragmentation, unclear roles and lack of accountability. Participants expressed that in ICR situations where the team is already at capacity, the team leader must decide which current member will be released to ensure optimal team composition. Furthermore, the intention should be to include the minimum number of team members. For effective interprofessional team collaboration the team leader needs to ascertain what unique value each healthcare professional will bring to the team and consider it when adding a team member. A particular challenge mentioned was that the team leader often changes in line with the composition of the team. This poses a risk of unclear leadership and affects team collaboration. Participants expressed;

“(…) it was jumbly and noisy in the room and many persons, a lot of concerns from midwives who asked ‘Should we all really be in here’? The physician then said ‘I don't know, if you don't think so, we will leave the room” (P1).

“It is always more difficult to have good communication when there are others around you talking. It is difficult to speak directly to the person you are trying to connect with” (P11).

#### Being prepared for intact umbilical cord resuscitation

This sub-theme highlights the importance of interprofessional team training and being prepared for ICR in the mother’s bed. Some participants had not participated in team training before exposure to an ICR situation and experienced a lack of control. The extensive number of team members in a small space reduced the control of the equipment and the overall view of the situation. Concerns such as how the equipment was positioned and how they gained access to the neonate were raised. The wellbeing and health of the neonate were considered the highest priority. Simultaneously, it was important to be able to fulfil the neonate’s need for closeness, warmth and attachment to the parents. To fulfil the neonate’s needs, it was crucial that interprofessional team collaboration was effective and timely. To ensure optimal solutions participants expressed a need to have the opportunity to train together in interprofessional teams. It was necessary to train positions, movements and equipment checks together in the labour room. Participants expressed that communication training was of significance for effective interprofessional team collaboration, as it reduced the risk of unclear division of responsibilities and roles, as well as conflicts. The participants perceived that debriefing after an ICR situation was essential to learn and be prepared for optimized interprofessional team collaboration. They expressed;

“I think all neonates should receive intact umbilical cord resuscitation. I think that's the optimal way to do it [neonatal cardiopulmonary resuscitation]. However, there are certainly several practical issues which need to be solved together such as how we get enough space, how we get access and how we fulfil the neonate’s need of warmth” (P2).

“It is important that you can collaborate during such a stressful situation in order to make it work, therefore it is vital that you have practised beforehand” (P11).

## Discussion

The aim of the present study was to explore neonatal healthcare professionals’ experiences of providing intact cord resuscitation in the mother’s bed. The results presented provide some insight into the participants’ experiences of the prerequisites for providing neonatal care in intact cord resuscitation and complement to the scarce research regarding healthcare professionals’ experiences of ICR. The most prominent result was how the healthcare professionals identified themselves with the mother who in their view was in a vulnerable position and situation.

The sense of the mother's vulnerability was noticeable, as the participants reported that they needed to protect and preserve the mother´s integrity by preventing exposure and maintaining dignity. Participants expressed that midwives were aware of the need to protect the mother from exposure and placed a towel over her abdomen. Nevertheless, the participants experienced the mother’s vulnerability, which may originate in identification with the mother’s situation. This could be an expression of the fact that they were not used to working close to a mother in a lithotomy position. Participants needed handling their emotions in an unfamiliar situation. On the other hand, they highlighted the benefits of providing ICR as neonates and parents were not separated. Bergman [20] highlights separation from the mother immediately after birth as a toxic stress for the neonate [20]. As birth involves two patients, namely mother and neonate, the starting point for the interprofessional team must be to avoid separating neonates and parents, thus promoting attachment between the mother and the neonate. Mothers expressed anxiety about being physically separated from their neonate who required care outside the delivery room, especially when there was unclear communication about where the neonate was taken [21].

The practical challenges in the environment included managing ICR in limited space and gaining access to the equipment needed for ICR. Due to an increased number of healthcare professionals and the equipment required for resuscitation, the labour rooms were often perceived as too small. Being unable to reach the equipment needed for resuscitation and observation of the neonate stressed the participants. Being the only representant in the profession can be experienced as lonely and vulnerable and it can be difficult to determine when to ask for help from one’s professional colleagues. A Swedish interview study with midwives indicates that feelings of being part of a team were important, as the team gave support in emotionally difficult and stressful situations [7]. The participants’ work experience increased their sense of security, hence where the resuscitation took place was not an issue. Physicians in obstetrics expressed that they felt lonely most of the time during obstetric emergencies, but those who had gained work experience invariably had both routines and know-how [22]. Therefore, it is important that all professionals involved in the ICR of the neonate have simulation training together to become a team.

The desire for interprofessional team training involved proper education and training as well as debriefing to manage ICR in the mother’s bed. Research indicates that team training is becoming increasingly important in healthcare contexts [13]. The healthcare organizations rely on teams to manage complex situations such as ICR. It is therefore essential that team members work together cohesively and effectively. Team training can help to address a range of issues that might arise, such as poor communication, lack of trust and conflicts between team members [23]. In addition, other challenges described in the literature were the integration of ICR into a clinical routine as well as team training and providing ICR to a non-vigorous neonate when witnessed by parents [13]. Team training can contribute to improved performance, increase job satisfaction, reduce stress and strengthen effectiveness and safe management by providing team members with the tools and techniques they need to work together effectively [24]. The interprofessional team members’ interpretation of *a shared mindset* indicated that the purpose of ICR was interpreted and valued differently based on their professions and specialties. This influenced interprofessional team collaboration. Thus, team training can foster a sense of companionship and a shared mindset including a common goal among team members [25]. Different types of team training can be effective depending on the needs of the team. Conflict resolution training including communication training can help team members to communicate more effectively, leading to fewer misunderstandings and efficient team collaboration. In addition, leadership training can assist team members to develop the skills they need to lead effectively, which is especially important in teams where there is no clear leader. Participants expressed that in ICR situations where the team is already at capacity, the team leader must decide which current member will be released to ensure an optimal team composition. A particular challenge expressed was that the team leader often changes in accordance with the composition of the team. Isacson et al. [7] highlighted midwives’ experiences of being a team member and participating in critical situations, *i.e.* an ICR situation. Negative feelings of insufficiency were expressed, which were caused by the midwives trying to balance different tasks at the same time. They wanted to calm the parents and explain what was happening, while simultaneously performing medical tasks required by the mother or neonate. Midwives balanced this by being a good team member and trying to consider the views of colleagues such as neonatal nurses, paediatricians, or obstetricians [7]. As ICR situations can be challenging in several ways, it is important to be vigilant about only adding team members when necessary. Too many team members can lead to miscommunication, confusion and unclear roles as well as a lack of effectiveness. It is therefore important to have a clear and concise plan of action as well as familiarity with guidelines. Participants expressed the need to be prepared for intact umbilical cord resuscitation together in the interprofessional team to have a shared mindset, thus preventing issues such as reduced control of the equipment, poor communication and conflict. In the literature, simulation training is a well-known method for increasing team members' competence and capacity in critical situations within healthcare organizations [26]. Debriefing is an essential part of simulation training, as it allows participants to reflect on their performance and identify areas for improvement. By discussing what went well and what could have been done differently, participants can develop a deeper understanding of the task at hand, improve interaction and be better prepared for future scenarios [23]. The analysis of data from the individual interviews had a qualitative inductive approach and some methodological considerations should be mentioned. One weakness in qualitative research is the lack of generalisability of the results. As qualitative research often involves a small sample size, the results may not be applicable to a larger population. However, the method used in the present study allowed in-depth exploration of a complex phenomenon, which involved collecting rich, detailed data from individual interviews. This provided a deeper understanding of the experiences and perspectives of participants, as well as the context of the study. A purposive sampling was used and the results represent 20 neonatal healthcare professionals’ experiences of providing ICR in the mother’s bed. The results are logical and congruent with the study aim. The formulated overarching theme *The prerequisites for providing neonatal care in intact cord resuscitation* run through the themes and sub-themes. In addition, the results were confirmed by using representative quotations in each sub-theme. Finally, there is a potential for subjectivity in the interpretation of the data. This is because qualitative research often relies on the researcher’s own interpretation of the data, which can be influenced by biases. It was therefore necessary to adopt an open approach to the interview text during the analysis process. In order to confirm the results, an outside expert (LTL) was asked to ensure the validity of the results and associated themes, *i.e.* achieve consensus.

## Conclusion

In conclusion, the result of the present study highlights that neonatal healthcare professionals’ experiences of providing ICR in the mother’s bed were positive and had significant benefits for the neonate, namely zero separation between the neonate and parents and better physical recovery for the neonate. However, the fact that ICR in the mother’s bed can be challenging in several ways, such as emotionally, managing environmental circumstances and ensuring effective team collaboration. Therefore, it is of the utmost importance that healthcare professionals are given the opportunity to reflect and train together as a team. Future recommendations are to summarize evidence-based knowledge to design guidelines for ICR situation.

## Data Availability

The datasets used and/or analysed during the current study available from the corresponding author on reasonable request.
